# The Processing of Negation and Polarity: An Overview

**DOI:** 10.1007/s10936-021-09817-9

**Published:** 2021-11-17

**Authors:** Carolin Dudschig, Barbara Kaup, Mingya Liu, Juliane Schwab

**Affiliations:** 1 Department of Psychology, University of Tübingen, Tübingen, Germany; 2 Department of English and American Studies, Humboldt University of Berlin, Berlin, Germany; 3 Institute of Cognitive Science, Osnabrück University, Osnabrück, Germany

**Keywords:** Negation, Polarity items, Conditionals, Questions, Language processing

## Abstract

Negation is a universal component of human language; polarity sensitivity (i.e., lexical distributional constraints in relation to negation) is arguably so while being pervasive across languages. Negation has long been a field of inquiry in psychological theories and experiments of reasoning, which inspired many follow-up studies of negation and negation-related phenomena in psycholinguistics. In generative theoretical linguistics, negation and polarity sensitivity have been extensively studied, as the related phenomena are situated at the interfaces of syntax, semantics and pragmatics, and are thus extremely revealing about the architecture of grammar. With the now long tradition of research on negation and polarity in psychology and psycholinguistics, and the emerging field of experimental semantics and pragmatics, a multitude of interests and experimental paradigms have emerged which call for re-evaluations and further development and integration. This special issue contains a collection of 16 research articles on the processing of negation and negation-related phenomena including polarity items, questions, conditionals, and irony, using a combination of behavioral (e.g., rating, reading, eye-tracking and sentence completion) and neuroimaging techniques (e.g., EEG). They showcase the processing of negation and polarity with or without context, in various languages and across different populations (adults, typically developing and ADHD children). The integration of multiple theoretical and empirical perspectives in this collection provides new insights, methodological advances and directions for future research.

Negation is a universal component of human language and polarity sensitivity (i.e., lexical distributional constraints in relation to negation) is arguably so while being pervasive across languages. Negation has long been a field of inquiry in psychological theories and experiments of reasoning, which inspired many follow-up studies of negation and negation-related phenomena in psycholinguistics. In generative theoretical linguistics, negation and polarity sensitivity have been a key field of study, as the related phenomena are situated at the interfaces of syntax, semantics and pragmatics. Formal and experimental research on these interface phenomena is thus of great importance towards gaining a better understanding of the architecture of grammar. The processes underlying the comprehension of negation and polarity have been investigated in both psychology and linguistics, using a combination of behavioral and neuroimaging techniques. Where do we stand now? This collection features 16 contributions from multiple theoretical and empirical perspectives, the integration of which provides new insights, methodological advances and directions for future research. In the following, we will provide a concise overview of the research topics dealt with in this collection, supplemented by a short summary of each contribution, in Section 1. Section 2 contains a short conclusion.

## Research Topics at Focus

**Fig. 1:**
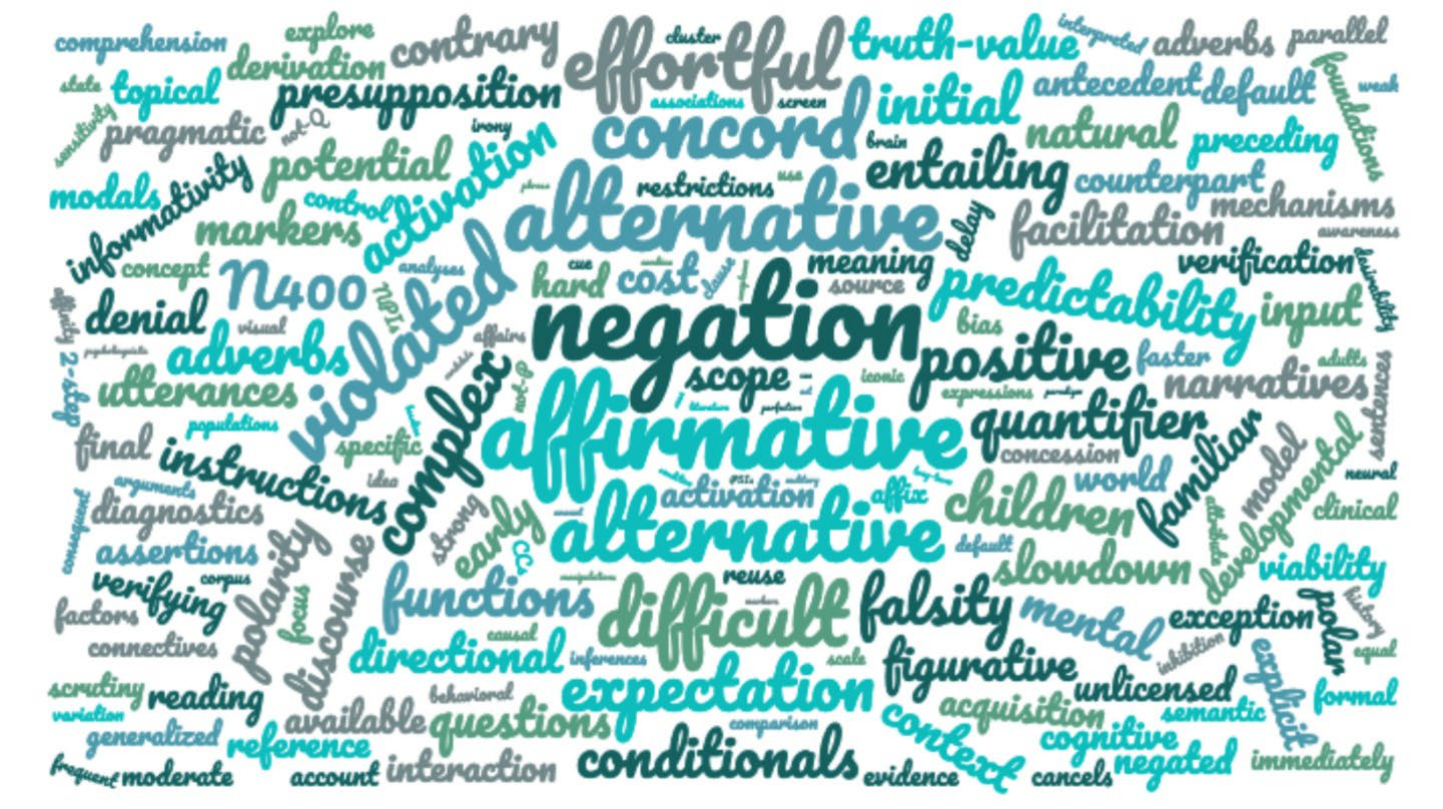
Word cloud based on the abstracts of all contributions to the Special Issue with slight modifications

The articles published in this special issue cover many different issues and research questions related to negation (see Fig. 1). We identified seven central topics which we will introduce separately in what follows.

### Negative Concord, Negative Quantifiers

In natural language, negation exhibits different forms and meanings which can match or mismatch. Canonical negation taking a negative form expresses a negative meaning. In contrast, negative concord or expletive negation is the cross-linguistically attested form/meaning mismatch whereby a negative quantifier or negative marker does not contribute to the meaning of proper (i.e., logical-semantic) negation. However, negative concord or expletive negation does not occur at random, and across languages, a large variety of triggers have been identified. There is an extant and growing body of theoretical literature on such non-canonical negation, however it remains far from clear how the negation involved in these structures is processed in comparison to single negation constructions.

**Maldonado & Culbertson** (2021) addresses the processing and learning of negative concord and double negation. They start with the observation that how negation is realized varies widely across languages. One of the factors in which languages show clear differences is how concurrent negative expressions *do* (or *do not*) interact with each other. In *double negation* languages such as Dutch or German each negative marker contributes a semantically independent negation, resulting in a doubly negated meaning. By contrast, in *negative concord* languages such as Serbian or French two concurrent negative expressions yield a single semantic negation. One of the longstanding questions in the linguistic literature concerns whether a language’s classification as double negation or negative concord language is systematically related to the properties of its negative markers. Jespersen (1917) originally put forward that all languages with phonologically strong markers of negation are double negation languages whereas all languages with phonologically weak markers are negative concord languages. This generalization has been reformulated and weakened by Zeijlstra (2004) to state that only languages that exclusively use affixes or particles as negative markers necessarily have to be negative concord. In their study, Moldonado and Culbertson use an artificial language learning task to investigate whether the Jespersen-Zeijlstra generalization is reflected in a cognitive bias in language learning. Using a miniature artificial language that employs either affixal or adverbial negation, they find that English speaking participants consistently find it easier to learn the artificial language as a negative concord than as double negation language. The status of the negative marker had no effect on the learnability of the language, contrary to the predictions from the Jesperson-Zeijlstra generalization. The authors discuss their findings in light of the characteristics of the artificial language that was employed and highlight the relation between their findings and the related literature on the processing and acquisition of negation.

### Negative and Positive Polarity Items

While negative concord constructions involve multiple negative expressions with a single negation interpretation (e.g., *not…nobody* in certain varieties of English), alternative constructions that give rise to similar meanings instead use negative polarity items (NPIs) (e.g., *not…anybody*). NPIs and their opposite positive polarity items (PPIs) require either the presence or the absence of negation or negation-like contexts. The nature of this requirement called ‘polarity sensitivity’ (syntactic, semantic, pragmatic) and the variation thereof (within and across languages) have been a topic in theoretical linguistic investigations. In related work on the processing of NPIs and PPIs, major questions in the field concern the cognitive mechanisms via which the licensing conditions are established, and potential differences (i) between licensing contexts of various negative strengths (e.g., sentential negation, downward-entailing operators, adversatives), (ii) between distinct statuses (NPIs vs. PPIs) and subclasses of polarity items, e.g., emphatic vs. attenuating NPIs, and (iii) between different age groups, aka the question of acquisition.

**Schaebbicke et al.** (2021) deals with the variation of NPIs and their licensing contexts in German, contributing to the questions in (i) and (ii). Their study is concerned with a large scale investigation of NPIs in German. While there is a vast literature of NPIs in both theoretical linguistics and psycholinguistics, most focus on a small set of them. This paper reports on two experiments. Experiment 1 tested the acceptability of 60 NPIs under semantic operators that are expected to license superstrong, strong, weak, and nonveridicality-licensed NPIs, including antimorphic (*nicht* ‘not’), anti-additive (*kein* ‘no’), downward entailing (*kaum* ‘hardly’), nonveridical (*vielleicht* ‘maybe’, questions), as well as a control affirmative condition. The results show that the tested NPIs cluster in seven groups, some of which are consistent with existing classifications in the literature, while others are not so easily accommodated. The most unexpected result is in cluster 4 where NPIs received high acceptability ratings in the antimorphic condition and in the question condition, and low ratings in all other conditions. In Experiment 2, the authors tested whether this is due to rhetoric use of questions licensing NPIs pragmatically, which they were not able to confirm. The large-scale study is a valuable contribution to the negation and polarity literature, in particular, with regard to the variation question, see Zwarts (1986, 1995, 1998), van der Wouden (1994), Giannakidou (1998), and Hoeksema (2012). The finding about the variation among individual NPIs and individual contexts as well as highly idiosyncratic licensing characteristics of NPIs enriches the empirical landscape of NPIs and raises further question in theoretical modeling.

**Schwab et al.** (2021) focuses on the L1 acquisition of German NPIs vs. PPIs, contributing to the questions in (ii) and (iii). Their study investigates the question how polarity items are acquired by looking at 11- to 12-year-olds’ comprehension of German NPIs and PPIs. PPIs and NPIs are restricted in their distribution to contexts of the appropriate polarity (Liu et al., 2019), but the complexity in the exact characteristics that render particular contexts *(anti-)licensing* and the considerable variability in individual polarity items’ sensitivity to such contexts poses a challenge to language learners. Previous research based on sentence production data (Lin et al., 2015, 2018; O’Leary & Crain, 1994; Tieu, 2013; Tieu & Lidz, 2016) suggest that children show an early sensitivity to the limited distribution of polarity items, although their knowledge of the set of licensing expressions grows more sophisticated with time. Schwab et al. extend the literature with a sentence judgment study on the comprehension of NPIs and PPIs in 11-12-year-olds. In testing four polarity items in total, they find that the tested children show adult-like responses for only one of them, while they tend to accept unlicensed polarity items to a higher extent than adults for the other three. The authors discuss their findings in the context of theoretical differences between emphatic and attenuating polarity items, and in relation to the neurophysiological literature on the timescale of language development, arguing that their findings are in line with research suggesting that the language system continues to mature throughout adolescence.

### Modals, Questions, Conditionals and Discourse Adverbs

Negation and negative quantifiers are not the only expressions that carry negativity. Rather, modals (e.g., verbs and adverbs), questions, conditionals, and certain discourse adverbs share with negation their status as entailment-cancelling contexts, such that the truth of the modified proposition under these operators is not entailed. Thus, although these contexts seem very different from canonical negation at the surface, they share crucial semantic properties (e.g., downward monotonicity or nonveridicality) with it, which also renders negation, questions, conditionals, etc. suitable licensers for NPIs and potential anti-licensers for PPIs. The processing of conditionals has been dealt with in psychological theories of reasoning. Modals, questions and discourse adverbs have traditionally been a field in linguistics, however, they have more recently been adressed in the processing literature as well. Liu & Barthel (2021), Orenes (2021) and Zevakhina & Prigorkina (2021) are all concerned with the processing of conditionals from different perspectives (i.e., in terms of logic-semantic vs. pragmatic meanings, symbolic vs. iconic representations and speech acts of promises vs. threats). Tian et al. (2021) reports experimental work on different kinds of polar questions. Liu et al. (2021) compares the processing of different kinds of conditionals in comparison with questions, with a focus on the effects of modal verbs and discourse adverbs.

**Liu & Barthel** (2021) explores the meaning and processing of German conditional connectives (CCs) such as *wenn* ‘if’ and *nur wenn* ‘only if’. While conditionals (e.g., *If it rains, the streets get wet.*) have been extensively studied in cognitive science, linguistics, logic, philosophy and psychology, there remain many open questions, one of which is concerned with how biconditionals are expressed (see the literature on *conditional perfection*, starting with Geis & Zwicky, 1971), and the related one of whether there are biconditional connectives in natural language. The paper reports on five sentence comprehension studies. Experiment 1, 2 and 5 focused on *wenn* vs. *nur wenn* ‘if vs. only if’: In Experiment 1, using a sentence completion task, participants read short scenarios containing a conditional sentence (i.e., If P, Q.) with *wenn/nur wenn* and a confirmed or negated antecedent (i.e., P/not-P), and subsequently completed the final sentence about Q (with or without negation). In Experiment 2, participants rated the truth or falsity of the consequent Q after reading a conditional sentence with *wenn/nur wenn* and a confirmed or negated antecedent (i.e., If P, Q. P/not-P. // Therefore, Q?). Experiment 5 used Affirmation of the Consequent (If P, Q. Q. // Therefore, P.) to test the (bi)conditionality of *wenn* vs. *nur wenn.* The results of the three experiments show that neither *wenn* nor *nur wenn* is semantically biconditional. Modus Ponens (If P, Q. P. // Therefore, Q) was validated for *wenn*, but not for *nur wenn.* While Denial of the Antecedent (If P, Q. not-P. // Therefore, not-Q.) and Affirmation of the Consequent were both validated for *nur wenn*, neither was validated for *wenn*. Taken together, the results provide first experimental and convergent supporting evidence for Herburger’s (2015, 2019) theoretical analysis of *only if*. The paper also reports on two more experiments, Experiment 3 and 4 testing (bi)conditionality of *unter der Bedingung, dass* ‘on condition that’ and *vorausgesetzt, dass* ‘provided that’, respectively. The results suggest that the former is the only and most likely candidate of biconditional CCs in German.

**Orenes** (2021) addresses the question how affirmative and negated compound sentences are processed by means of a visual world paradigm. Participants listened to orally presented sentences which could be classified as affirmative (“Because he was hungry, he ordered a salad”) vs. negative causal assertion (“Because he was not hungry, he did not order a salad”) or affirmative (“If he had been hungry, he would have ordered a salad”) / negative counterfactual (“If he had not been hungry, he would not have ordered a salad”). On a screen four options were displayed, i.e., “salad”, “no salad”, “soup” and “no soup” and participants’ eye movements across these options were recorded during listening to the critical sentences. The main findings indicated that participants look at the explicit negation “no salad” rather early, especially for negative causal assertions, but rather late at the potential alternative “soup”. The author makes the following conclusion from this data: indeed, the symbolic negation itself is integrated rather early in processing, however, the process that takes time is the representation of a potential alternative and the representation of the alternative is not required to process negation. The author interprets the data with relation to various accounts of negation processing (e.g., embodied, pragmatic account). The article also suggests that given the limitations of the visual world paradigm with regard to online negation processing, some conclusions need to be investigated further in future studies.

**Zevakhina & Prigorkina** (2021) deals with the processing of conditionals with a focus on conditional perfection in different speech acts. Conditional perfection (Geis & Zwicky, 1971) refers to the pragmatic phenomenon whereby conditional sentences of the form *If p, then q* may receive a biconditional interpretation of the form *If and only if p, then q*. Conditional perfection is a widely studied topic in logic, linguistics, and psychology, with the focus of the present paper being on two particular conditional speech acts: As Evans and Twyman-Musgrove (1998) suggested that conditional promises and threats are particularly likely to trigger perfected interpretations, Zevakhina and Prigorkina set out to investigate the derivation of conditional perfection in promises and threats under a range of linguistic modifications. They conducted two inference and reaction time experiments which varied (i) the listener’s incentive to collect the promised or avoid the threatened outcome, (ii) the order of antecedent and consequent, and (iii) the presence of negation in either antecedent or consequent. Regarding the overall rate of conditional perfection, both experiments fail to provide evidence for a difference between promises and threats, a difference depending on the order of the antecedent and consequent proposition, or an effect of incentive. Experiment 1, however, does indicate that promises and threats differ with regard to the effect of negating the antecedent or consequent proposition: In threats, conditionals with a negated consequent yielded higher rates of conditional perfection with accompanying longer reaction times, while in promises conditional perfection was not affected by the presence of negation. The authors argue that their results on threats align with previous research, while their findings on conditional promises suggest a difference between the two conditional speech acts. On a broader scope, they conclude that their findings show that conditional perfection is not an effortful inference, supporting accounts of conditional perfection such as van Tiel & Schaeken (2016).

**Tian et al.** (2021) investigates the processing of polar questions in English and French. The linguistic properties of polar questions have been extensively studied (see Groenendijk & Stokhof, 1984; Hamblin, 1973; Krifka, 2001; Roelofsen & Farkas, 2014, among many others), however, how polar questions are processed in real time remains unclear. This paper presents three visual world eye-tracking experiments investigating how polar questions are processed and how this processing is affected by their form (positive, high-neg, or low-neg in English, e.g., *Has Anne closed her mom’s umbrella?/Hasn’t Anne closed her mom’s umbrella?/Has Anne not closed her mom’s umbrella?*). In the experiments, participants are presented with visual scenes containing relevant objects while listening to linguistic stimuli (e.g., Breheny et al., 2013; Tian et al., 2016). In Experiment 1, participants listened to these question-answer pairs, and answered comprehension questions to ensure that they were paying attention. While listening, participants saw four images: the p image, the ¬p image, and two distractor images. In Experiment 2, participants had to select the right display based on the answer to the question. Experiment 3 tested positive and negative polar questions in French. Overall, the authors observed positive biases in positive polar questions and to a lesser extent in high-neg questions in English and negative questions in French, whereas English low-neg questions showed no clear bias. They conclude that the different biases in mental representations reflect the hearer’s reasoning about the speaker’s purposes of enquiry.

Finally, **Liu et al.** (2021) considers speaker assumptions about the truth of the antecedent proposition in conditionals. Epistemic modals, questions, and conditionals are nonveridical (or in other words, entailment-cancelling) contexts like negation (Giannakidou, 1998). Following the recent work of Giannakidou & Mari (2021), the authors assume “nonveridical equilibrium” (implying that p and ¬p as equal possibilities) to be the default for epistemic modals, questions and conditionals, but argue that the equilibrium can be manipulated to produce bias. They report on two rating experiments in German with a focus on the adverb *wirklich* ‘really’, the modal verb *sollte* ‘should’ and conditional connectives such as *falls* ‘if/in case’ (see Liu 2019, 2021; Reis & Wöllstein 2010). Experiment 1 tested whether the presence of *wirklich* or *sollte* lowers the ratings of the speaker commitment toward the antecedent proposition, as well as whether questions and conditionals led to similar or different ratings. The results show that questions (*ob*-p ‘whether p’) received significantly lower ratings than conditionals (*wenn-*p ‘if p’) as well as the commitment-reducing effect of both *wirklich* and *sollte.* Experiment 2 tested the effect of *wirklich, sollte*, and different kinds of conditionals (*wenn, falls*, V1 —verb first, e.g., *Werde ich Anwältin, mache ich eine eigene Anwaltskanzlei auf.* ‘become I lawyer, make I one own law firm open, i.e., If I become a lawyer, I will open a law firm of my own.’). It replicates the results of *wirklich* and *sollte* in Experiment 1. Furthermore, the results show that *falls*-conditionals express a lower speaker commitment about the modified (antecedent) proposition than *wenn-*conditionals (in line with the findings of Liu, 2021) and V1-conditionals. This study shows that conditionals can be manipulated to produce bias as in biased questions or modalized declaratives, and provides a first step for the empirical domain of conditionals and bias.

### Difficulty of Negation Processing and the Activation of the Affirmative State of Affairs

In psycholinguistic research on negation processing, one of the central points of debate concerns the question whether or under which conditions negation is more difficult to process than affirmation, and the related issue of whether negative meaning is processed immediately (i.e., the one-step model of negation processing), or has to be computed from the affirmative content in a two-step model of negation comprehension. While out-of-the-blue negation has been associated with increased processing costs, it has been argued that pragmatic factors mitigate this cost by providing a licensing context for negative utterances. The contribution by Albu et al. (2021) focuses on the question whether there are certain contexts in which negation is as easy to process as affirmation. The contribution by Clifton et al. (2021) presents experimental studies that investigate predictions relevant to the question whether the affirmative portion of the sentence (i.e., the negated state of affairs) plays a special role for the comprehension of negation.

**Albu et al.** (2021) addresses the question whether and how context influences the processing of negation. Out of context, negative sentences are often more difficult to process than their affirmative counterparts (for review, see Kaup & Dudschig, 2020). In so-called “contexts of plausible denial” or in contexts where negation is pragmatically licensed (in terms of the relevance or informativity of the negative utterance), however, this processing cost is reduced or even eliminated (Nieuwland & Kuperberg, 2008; Tian et al., 2010; Xiang et al., 2020; *among others*). Albu et al. conducted six behavioral experiments to investigate the extent to which linguistic markers of plausible denial, such as *contrary to expectations*, can reduce the processing cost associated with negation. In the first four experiments, they show that preceding a negative sentence with the cue phrase *contrary to expectations* does not reduce its processing cost in either response times on a sensibility judgment task (Experiments 1 and 2) or reading times in a self-paced reading task (Experiments 3 and 4). By contrast, if the negative sentence is preceded by a linguistic context in which the expectation to be denied is mentioned explicitly (Experiment 5) or in which the phrase *contrary to expectations* follows an extended narrative (Experiment 6), the processing of negation is facilitated. On the basis of their findings, the authors conclude that minimal linguistic contexts of plausible denial are not sufficient to reduce the processing cost of negation. Instead, the processing difficulty of negation in written contexts is largely governed by pragmatic factors.

**Clifton et al.** (2021) presents three reading and rating experiments on ellipsis and *as*-clauses in English. While negative sentences arguably presuppose or implicate an affirmative counterpart and their processing has been shown to be faster in contexts with corresponding information (see Kaup & Dudschig, 2020), the question arises for elliptical structures under negative sentences whether there is a preference for affirmative antecedents over negative ones. Against this background, Experiment 1 tested whether the *as-*clause adjective (e.g., *Don’t cross on red as a stupid/smart person would.*) and the presence or absence of a comma before the *as*-phrase (e.g., *Don’t cross on red, as a stupid/smart person would.*) had an effect on acceptability ratings and reading times. The results show that the sentences with the non-desirable attribute (“stupid”) were read faster and rated more acceptable than the ones with the desirable attribute (“smart”). The comma, which blocks low attachment (see Rayner & Frazier, 1987), did not have an effect, which rules out the possibility that the found effect was due to low attachment preferences. Experiment 2 tested the acceptability of sentence continuations, e.g., *Don’t cross on red, as a stupid/smart person would. He probably would end up hurt/safe.* Among others, the results showed the predicted interaction between the desirable/non-desirable attribute in the *as*-clause and the adverb in the continuation: In the case of the non-desirable attribute (“stupid”), the continuation involving “hurt” was judged to be more acceptable than the continuation involving “safe”, whereas in the case of the desirable attribute (“smart”), both continuations were judged to be approximately equally (un)acceptable. Experiment 3 showed that the effects generalized to non-imperative (declarative) sentences. Overall, the authors take the results to indicate that *do*-ellipsis picks up positive antecedents more readily than negative antecedents.

### Interaction between Polarity and Truth Value

Another issue of debate is related to the often-observed interaction of negation and truth value. While affirmative sentences are easier to process when they are true rather than false, the same does not hold for negative sentences. In the literature on negation processing, several factors have been suggested to underlie this interaction, namely, lexical associations, pragmatic factors and predictability. So far conclusive evidence is still lacking. The contributions by Rück et al. (2021) and Wang et al. (2021) focus on the interaction of negation and truth value. We provide short summaries of the two papers below.

**Rück et al.** (2021) addresses the question how the effect of polarity (negation/affirmation) and the effect of truth value (true or false) interact with each other in processing and what role predictability plays for the occurrence of this interaction. Concerning the known observation about the interaction between polarity and truth value, there are two potential explanations (i.e., lexical associations and predictability) for the processing difficulty for true-negative sentences (e.g., “Zebras are not dotted”) in comparison to false-negative ones (e.g., “Zebras are not stripy”), as documented in the literature (see among others, Carpenter & Just, 1975; Clark & Chase, 1972; Nieuwland, 2016). The authors use a behavioral paradigm to investigate the processing of true and false negative sentences in contexts controlled for lexical associations with varying predictability. In the experiment, participants saw “artificial worlds” of geometric shapes followed by a corresponding sentence and were asked to decide as quickly as possible whether the sentence was true or false regarding the presented picture, while reaction times and accuracy were recorded. The results show two main effects of polarity and truth value, as well as an interaction of the two factors. Based on this, the authors argue that long-term lexical associations are not responsible for the observed interaction, which would have only predicted two main effects but no interaction effect. Furthermore, they also found an effect of predictability in that the truth-value-by-polarity interaction was more visible in the high-predictable conditions than in the low-predictable conditions. This casts doubt on the validity of the explanation relying on predictability as suggested by Nieuwland (2016), which would predict a truth-value-by-polarity interaction in particular in the low-predictable conditions in the given experiment.

**Wang et al.** (2021) addresses the question under which conditions sentence verification tasks involving true and false affirmative and negative sentences result in a main effect of truth value versus a truth value by polarity interaction. Response times and accuracy rates in sentence verification tasks often exhibit a truth value by polarity interaction in the sense that, for affirmative sentences, true versions are faster and more accurate to verify than false versions whereas the same is not true for negative sentences. Here false versions are either equally fast and accurate as true versions or even more accurate and faster. Typically, this result pattern is taken as reflecting a two-step procedure of verification of negative sentences, in which the positive counterpart is being verified and the result is then reversed from true to false and vice versa. However, sometimes sentence verification tasks also lead to a main effect of truth value, reflecting faster and more accurate responses for true compared to false versions independent of polarity. This result pattern is usually taken as reflecting a direct interpretation of negative sentences in only one step. In their study Wang et al. investigate the prediction that readers tend to employ a two-step procedure only in default contexts (cf. Tian et al., 2010). For default contexts, the authors presented polar questions (*Is the apple pealed?*) with an affirmative or negative answer (*It is* / *It isn’t*) plus a picture that either matched or mismatched the given answer to the question (e.g., a peeled apple and an unpeeled banana). For non-default contexts, the authors presented affirmative and negative wh-questions (*Which one is/isn’t peeled?*) with a noun phrase as answer (e.g., *The apple* vs. *The banana*) plus a matching or mismatching picture. As predicted, accuracy rates and response times showed a truth value by polarity interaction only in the default contexts but a main effect of truth value in the non-default contexts, supporting the idea that a two-step procedure is not mandatory in sentence verification and emphasizing the role of the context in sentence verification involving negation.

### Speech Acts, Discourse Functions and Figurative Use

Negation and polarity phenomena give rise to speech acts (e.g., denial and rejection), discourse functions (e.g., concession) and can be used figuratively (e.g., irony and understatement). The contributions of Albu et al. (2021; see above) and Crible (2021) focus on the effect of discourse relations for negation, and the contribution of Filik et al. (2021) investigates the processing of ironic statements in order to validate the presupposition-denial account.

**Crible** (2021) changes the focus of research from investigating what factors make negation easier to process to the question what is made easier by negation. Previous research has shown differences in the ease with which different discourse relations are processed. For instance, result relations, which typically express an expected link between two events (“My sister is a bad cook and so the desert she made was disgusting”), are easier to process than concession relations, in which the second segment is unexpected and typically denies a causal inference from the first segment (e.g., “My sister is a bad cook but the dessert she made was delicious”; e.g., Morera et al., 2017; Xu et al., 2018). When the first segment of the concession is negative, the denial of expectation inherent to concession is made explicit, possibly a facilitating factor in the processing of concessive discourse relations. Indeed, corpus studies have shown that negation frequently occurs in the context of concession relations (Asr & Demberg, 2015). In four self-paced reading experiments, Crible investigated the question whether negation provides a cue to better integrate a concession relation. Participants were presented with affirmative and negative segments followed by a result or concessive relation (e.g., “The students knew / didn’t know their course work and they were confident/anxious about their performance”) and were asked to judge whether the sequences made sense or not. An analysis of the reading times showed that indeed concessive relations were harder to process than result relations in affirmative conditions whereas this difference was diminished when a negation was present in the first segment. The authors conclude that indeed negation may be a cue for an upcoming concession relation and thus may facilitate its processing.

**Filik et al.** (2021) tests whether positive quantifiers when used in an ironic context mimic negative quantifiers in licensing complement set reference. Negation when used for denying a presupposition implies a deviation between what is expected and what is actually the case. Negative quantifiers in particular imply a shortfall between a factual amount and a larger expected amount. According to the Presupposition-Denial Account, this is the reason why complement set reference often arises with negative quantifiers (e.g., *Few fans came to the football match. They watched it on TV instead*; cf. Moxey et al. 2001) but usually not with positive quantifiers which do not imply such a short fall (e.g., A few fans came to the football match. *They watched it on TV instead). However, when used in an ironic manner, positive quantifiers may also be used to highlight the shortfall between what is expected or desired and what is the case. In their study the authors investigated whether positive quantifiers used ironically would also license “complement set reference”, as predicted by the Presupposition-Denial account. In line with their hypotheses, ERPs during reading complement set references indicated a smaller N400 following an ironic compared to a non-ironic use of a positive quantifier, providing evidence that the shortfall implied through irony is sufficient to license complement set reference.

### Inhibitory Mechanisms

It has been argued that negation relates to cognitive inhibitory mechanisms, showcasing an additional important strand of research on negation crossing the linguistic and non-linguistic domain. Beltrán et al. (2021) provides a review of empirical studies investigating the role of inhibitory mechanisms for negation and Dudschig et al. (2021) investigates this issue in a population of neurotypical children and children with an ADHD diagnosis.

**Beltrán et al.** (2021) comprehensively summarizes all empirical studies that support such an interrelation between linguistic and non-linguistic cognition specifically with regard to negation processing. Such an overarching view of negation processing—if it holds further tests—is not only relevant within the field of negation processing—but also for understanding the human cognitive architecture as a whole, as it would support the broader neural reuse hypothesis (Anderson, 2010). The neural reuse hypothesis suggests that during evolution or development neural circuits that serve a similar function can be shared via different mechanisms for different purposes. The main support for such a neural reuse principle in the case of negation processing currently stems from behavioral and electrophysiological evidence, showing an intercoupling between inhibitory processes relevant in action control and negation processing. Nevertheless—as for all rather new hypotheses—the article also points out that this idea of a generally shared inhibitory network between linguistic negation and action control would need further empirical support. For example, it is still open whether the involvement of such inhibitory networks during negation processing is indeed functionally relevant for comprehending negation, or whether such associations can be found beyond dual-task paradigms and whether they apply for a wide range of negation types.

**Dudschig et al.** (2021) reports a pilot study aimed to assess how neurotypically healthy and ADHD children process negated instructions. Recent literature suggests that negation comprehension falls back onto inhibitory brain systems that are also crucial for impulse control and other non-linguistic control domains (Beltrán et al., 2018, 2019; de Vega et al., 2016; Liu et al., 2020). In the reported pilot study, the use of negation within directional instructions (i.e., “not left”, “now left”, “not right”, “now right”) was tested in children with ADHD and an age-matched control group. Slower responses were found for negative relative to affirmative instructions, with the discrepancy being larger in the ADHD group than in the control group. The authors concluded that understanding negation calls for inhibitory control processes which are differently available across different subgroups. While negation in imperatives or instructions needs to be used carefully due to potential processing costs, negation use in specific clinical populations might have to be handled with even more care. Thus, the paper is not only of theoretical relevance but also of practical significance, which calls for more extensive investigation of negation use in different populations.

## Conclusions

With the now long tradition of research on negation and polarity in psychology and psycholinguistics, and the emerging field of experimental semantics and pragmatics, a multitude of interests and experimental paradigms have emerged which call for re-evaluations and further development and integration. This special issue comes in timely.

The 16 research articles address negation and various negation-related phenomena including polarity items, questions, conditionals, and irony (see Fig. 1). They showcase the processing of negation and polarity with or without context and in various languages. While many of these relate to extant research areas, they present new perspectives on old data, as well as new data and new insights on the structure, meaning (semantics and pragmatics), processing and even application of negation and polarity. In addition, whereas most studies are concerned with healthy adults, some compare adults with typically developing or ADHD children, connecting to research on acquisition and disorders. Moreover, the special issue includes studies in artificial language as well as in connection with cognition outside of the linguistic domain—both pointing at future avenues of research on negation and cognition.

Methodologically, the reported studies apply a combination of behavioral (e.g., rating, reading, eye-tracking) and neuroimaging techniques (e.g., EEG), which is useful not only for studying negation and polarity, but also for studying meaning, cognition and processing in general.
